# Prognostic Tools for Older Women with Breast Cancer: A Systematic Review

**DOI:** 10.3390/medicina59091576

**Published:** 2023-08-30

**Authors:** Sydney M. Record, Tori Chanenchuk, Kendra M. Parrish, Samantha J. Kaplan, Gretchen Kimmick, Jennifer K. Plichta

**Affiliations:** 1 Department of Surgery, Duke University Medical Center, Durham, NC 27710, USA; 2 Medical Center Library, Duke University, Durham, NC 27710, USA; 3 Duke Cancer Institute, Duke University, Durham, NC 27710, USA; 4 Department of Medicine, Duke University Medical Center, Durham, NC 27710, USA; 5 Department of Population Health Sciences, Duke University Medical Center, Durham, NC 27710, USA

**Keywords:** prognostic tools, older, elderly, survival, prognosis

## Abstract

*Background*: Breast cancer is the most common cancer in women, and older patients comprise an increasing proportion of patients with this disease. The older breast cancer population is heterogenous with unique factors affecting clinical decision making. While many models have been developed and tested for breast cancer patients of all ages, tools specifically developed for older patients with breast cancer have not been recently reviewed. We systematically reviewed prognostic models developed and/or validated for older patients with breast cancer. *Methods*: We conducted a systematic search in 3 electronic databases. We identified original studies that were published prior to 8 November 2022 and presented the development and/or validation of models based mainly on clinico-pathological factors to predict response to treatment, recurrence, and/or mortality in older patients with breast cancer. The PROBAST was used to assess the ROB and applicability of each included tool. *Results*: We screened titles and abstracts of 7316 records. This generated 126 studies for a full text review. We identified 17 eligible articles, all of which presented tool development. The models were developed between 1996 and 2022, mostly using national registry data. The prognostic models were mainly developed in the United States (*n* = 7; 41%). For the derivation cohorts, the median sample size was 213 (interquartile range, 81–845). For the 17 included modes, the median number of predictive factors was 7 (4.5–10). *Conclusions*: There have been several studies focused on developing prognostic tools specifically for older patients with breast cancer, and the predictions made by these tools vary widely to include response to treatment, recurrence, and mortality. While external validation was rare, we found that it was typically concordant with interval validation results. Studies that were not validated or only internally validated still require external validation. However, most of the models presented in this review represent promising tools for clinical application in the care of older patients with breast cancer.

## 1. Introduction

Breast cancer is the most commonly diagnosed cancer with ~300,000 new diagnoses in 2022, and it is the second leading cause of cancer death among women worldwide [1]. Preventative screening through mammography as well as the incorporation of recent treatment advances into care can prevent death and improve outcomes. As the population ages, elderly patients comprise an increasing proportion of patients diagnosed with breast cancer. However, older patients with breast cancer are heterogenous. Some elderly patients will have relatively indolent tumors while others will not be diagnosed until the tumor has resulted in distant metastases [2,3]. Often, due to increased comorbidities and perceived poor treatment tolerance, elderly patients may receive less adjuvant therapy [4]. Given the variable nature of elderly patients and their diagnoses, evidence for and guidance on when a patient will benefit from adjuvant treatment is lacking.

A prognostic model taking into consideration unique factors, such as frailty and comorbidities, to predict the individual risk of recurrence, progression, and clinical outcomes for elderly women with breast cancer is critical to help guide treatment decisions in the clinical setting. These prognostic tools can assist clinicians in determining the best route of treatment for elderly patients. A prognostic model utilizes a statistical equation to evaluate multiple predictors from which the risk of a specific outcome can be determined on an individual basis [5]. Several prognostic models are currently used in the breast cancer field, but few are specifically tailored to the elderly population. In order to assist physicians and elderly patients with treatment decisions, there is an urgent need to systematically summarize prognostic tools and assess their performances.

In this study, we conducted a systematic review to identify and summarize prognostic models for determining treatment options in older women with breast cancer. The prognostic models were evaluated based on the guidelines of the Checklist for critical appraisal and data extraction for systematic reviews of prediction modeling studies (CHARMS) and the prediction model risk of bias assessment tool (PROBAST) in order to determine the validity and reliability of models and provide a framework to guide clinicians and future research in this field [6,7].

## 2. Methods

### 2.1. Information Sources and Search Strategy

Each step of this systematic review was conducted in accordance with the PRISMA 2020 statement [8]. A medical librarian with expertise in systematic searching composed a sensitive search utilizing a mix of subject headings and keywords to represent the concepts of elderly adults, breast cancer patients, and tools. The search was peer reviewed by another medical librarian according to a modified PRESS checklist. The databases MEDLINE via PubMed, Embase via Elsevier, and the Web of Science Core Collection (1900-present) via Clarivate were searched from inception until 8 November 2022. When possible, non-human studies and conference abstracts were removed. All results were compiled into EndNote and imported into Covidence for screening and deduplication. All search strategies are detailed and available in Appendix A. The protocol was also registered and can be accessed in the PROSPERO database (protocol number 448967).

### 2.2. Eligibility Criteria

We included studies if they reported the development, update, or external validation of at least one multivariable prognostic model based on individual characteristics, and the outcome of the prognostic model was a clinical outcome (e.g., response to a specific intervention, recurrence, disease-specific, or overall survival) in older patients diagnosed with breast cancer.

Studies were excluded if they (1) were not specific to older women with breast cancer; (2) were conference abstracts, letters, editorials, or non-original studies, such as reviews; (3) focused on methodology; (4) developed or validated diagnostic or screening models; (5) did not construct a model to estimate individual risks; and (6) were not in English.

### 2.3. Study Selection

Study selection was conducted using Covidence (Melbourne, Australia). Duplicates were found and removed automatically and manually. Two independent reviewers (authors S.M.R. and T.C.) with backgrounds in breast oncology and uniform training performed preliminary screening of titles and abstracts using pre-defined eligibility criteria. Any disagreement between reviewers was resolved by discussion and consensus.

### 2.4. Data Extraction

Two reviewers (authors S.R. and T.C.) extracted information from each relevant publication through a standardized form based on the CHARMS checklist [6]. The items extracted from each primary study were grouped within 11 domains, including source of data, participants, outcome(s) to be predicted, candidate predictors (or index tests), sample size, missing data, model development, model performance, model evaluation, results, interpretation, and discussion. Data extraction also included basic characteristics, including title, author, publication year, and whether the study was a development or validation study.

The included models were divided into 3 groups based on the type of prediction made by the tool: benefit from a specific clinical intervention, frailty, or mortality.

### 2.5. Quality Assessment

Each model was assessed independently by 2 reviewers (authors S.M.R. and T.C.) for risk of bias (ROB) and applicability. The PROBAST was used to assess the ROB and applicability of each included tool [7]. This tool utilizes 4 domains (participants, predictors, outcome, and analysis) for assessment.

Assessment of ROB and applicability utilized 20 signaling questions concerning the key aspects of prognostic models, which could be answered as “yes”, “no”, or “no information” by each reviewer. The answers were used to judge the ROB of each domain as “low”, “high”, or “unclear”, according to indications outlined by PROBAST. Per this tool, a study was judged as low ROB if all domains were judged as low risk and high risk if one or more domain was judged as high ROB. Similarly, answers were used to judge the applicability as “low”, “high”, or “unclear” for each domain and overall using PROBAST guidelines.

### 2.6. Data Synthesis

This study consisted of descriptive analyses of the characteristics of the identified prognostic models. Reported characteristics include date of publication, minimum patient age, number of patients in the development cohort, source of data for the development cohort, number of patients in the validation cohort, and source of data for the validation cohort. Presence or absence of validation was reported, including relevant validation statistics, namely the C-Index (Concordance Index) and AUC [Area Under the Receiver Operating Characteristic (ROC) Curve]. The ROC is a probability curve, and the AUC represents the degree to which it is capable of distinguishing between outcomes. The C-Index is a generalization of the AUC that also accounts for censored data. In addition to the above reported measures, models were grouped by type of prediction and presented with eligibility criteria and predictive factors utilized.

Due to the heterogeneity of study designs, predictive factors, inclusion criteria, model outcomes, measurement techniques, and methods of analysis, meta-analysis was not undertaken.

## 3. Results

**Figure 1:**
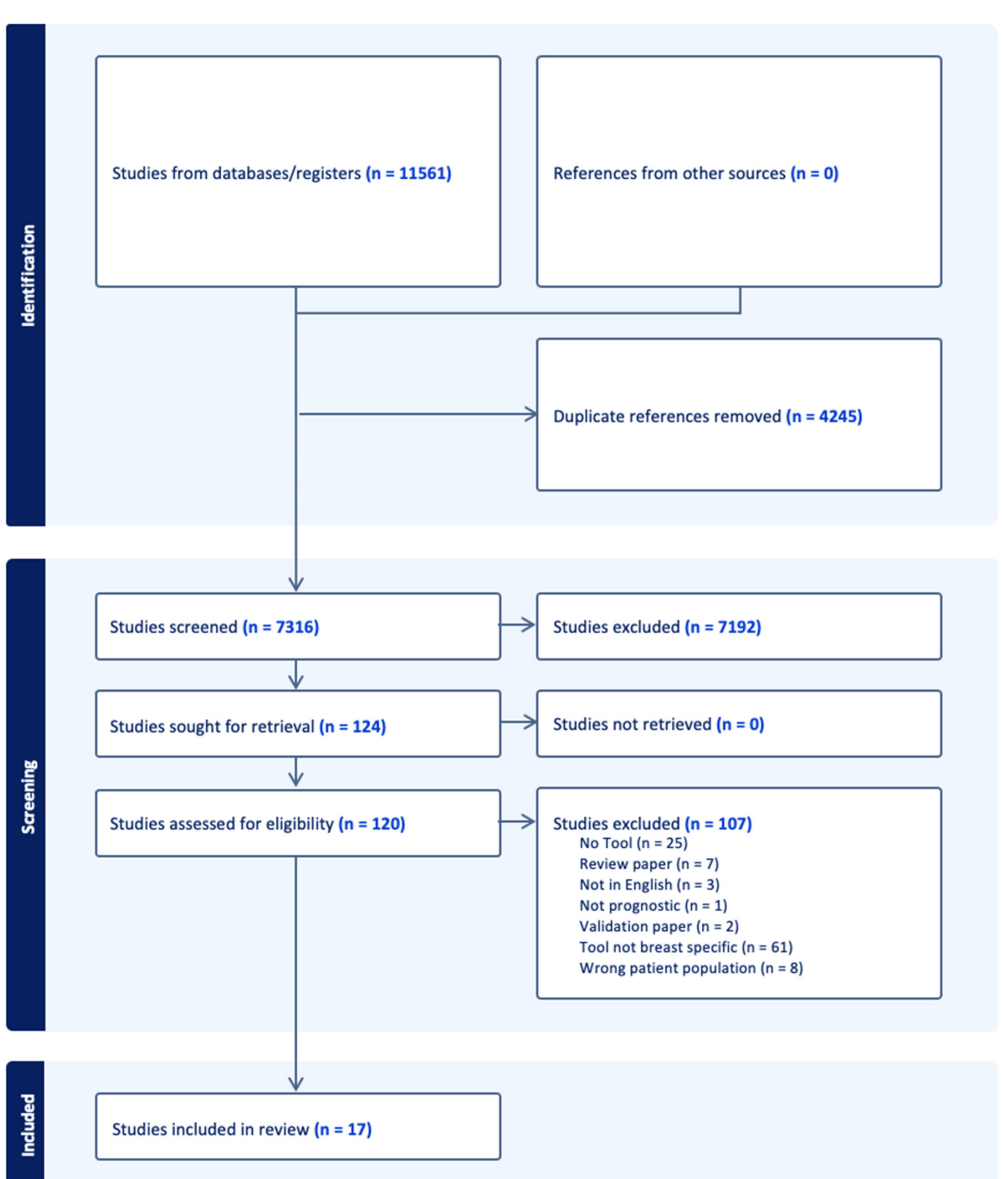
Flow diagram of literature search process for a systematic review of prognostic tools for older patients with breast cancer.

The systematic search in the 3 databases generated 11,437 records (Figure 1). We excluded 4245 duplicates. We screened the titles and abstracts of the remaining 7316 records. This generated 126 studies for a full text review. We identified 17 eligible articles, all of which presented tool development. Additionally, 6 articles presented internal validation only and 5 articles presented both internal and external validation. One article presented data regarding the implementation of a tool [9].

The models were developed between 1996 and 2022, mostly based on retrospective analyses of national registry data (Table 1). The prognostic models were mainly developed in the United States (*n* = 7; 41%). For the derivation cohorts, the median sample size was 213 (interquartile range, 81–845). For the validation cohorts, the median sample size was 4443 (1458.5–14,096.5). For the 17 included models, the median number of predictive factors was 7 (4.5–10; Table 2).

### 3.1. Prognostic Models for Adjuvant Radiation

Four models were developed for older breast cancer patients receiving adjuvant radiation therapy. All of these models had a sufficient sample size and predicted the benefit of receiving radiation therapy (RT) on the basis of various factors. However, three out of the four models have not yet been validated, and none have been externally validated, making their prognostic capabilities somewhat uncertain.

Albert et al. developed a nomogram that can be used to estimate mastectomy-free survival and predict the benefit of radiation for patients treated with breast conserving surgery (BCS) based on age, race, tumor size, estrogen receptor (ER) status, RT, and nodal pathology [10]. This model was developed using data from 16,092 women aged 66–79 in the Surveillance, Epidemiology, and End Results (SEER) program. Internal validation showed moderate predictive ability, with a C-index of 0.655.

Chen et al. created a nomogram predicting the benefit of RT on 10-year cancer specific survival following breast conserving therapy in patients 70 years and older with stage I and ER-negative or stage II/III disease [11]. Benefit of RT was predicted based on ER and progesterone receptor (PR) status, tumor grade, T stage, N stage, and race using data from 9079 patients in the SEER database. The Hosmer–Lemeshow test showed that the nomogram was well calibrated (*p* = 0.99). Internal validation was performed by examining discrimination, meaning whether the patients predicted to have a specific outcome are those who did, using receiver operating characteristic (ROC) curve (AUC). After adjustment, the AUC was 0.679 [11]. However, the model still needs to be validated using an external dataset and does not consider other important factors, such as human-epidermal-growth-factor-receptor-2 (HER2) status, comorbidities, or life expectancy.

ROW (Radiotherapy for Older Women) is a risk calculator for older women with early-stage breast cancer that can be used to estimate individualized probability of long term survival and local recurrence developed by Wang et al. [12]. Rather than real patient data, the investigators utilized 56,700 simulations. They used two existing prediction models (Breast Cancer trialists collaborative group prediction model and ePrognosis) to construct a Markov model, integrating 10-year mortality as a background, to develop ROW. ROW considers 16 predictive factors, including age, tumor grade, tumor size, ER status, and RT receipt, in lymph-node negative women who undergo BCS. However, ROW is limited in that simplified approximations were used, and the tool has yet to be validated. If validated, the risk calculator could be used both in clinical practice as well as be integrated into a larger decision-support tool. 

Abujarad et al. developed ROW into a user-friendly digital health risk calculator to estimate local cancer recurrence and all-cause mortality with and without RT [9]. There were 28 participants, all aged ≥65, at a single institution. Participants had a high level of satisfaction with this tool; however, a homogenous patient population was used, and its acceptability among clinicians was not tested [9].

A more focused study was performed by Rades et al., who created a prognostic instrument to estimate the overall survival benefit of RT in patients age ≥ 65 receiving RT for breast cancer metastasized to the spinal cord [13]. This model was developed using data from 218 patients from a single institution. The instrument factors in age, time from diagnosis to RT, fractionation regimen, metastatic status, ambulatory status pre-radiation, and the Eastern Cooperative Oncology Group performance score. This predictive instrument has the potential to contribute significantly in this distinct area of breast cancer care; however, it has not been validated internally or externally.

### 3.2. Prognostic Models for Patients Undergoing Chemotherapy

Magnuson et al. developed a tool, the Cancer and Aging Research Group-Breast Cancer (CARG-BC) score, by utilizing data from 283 patients in the Hurria Older PatiEnts (HOPE) with Breast Cancer Cohort Study [14]. HOPE was a multi-institutional prospective study of postmenopausal women with stage I–III breast cancer on neoadjuvant or adjuvant chemotherapy. CARG-BC is a tool to predict severe toxicity events for older patients receiving adjuvant or neoadjuvant chemotherapy. It uses eight predictive factors including age, sex, race, household composition, planned treatment regimens, and geriatric assessment (GA) variables. The model was internally validated and externally validated using data from an additional 190 patients in the HOPE cohort. CARG-BC demonstrated good discrimination in both, with an AUC of 0.75 internally, 0.69 externally, and 0.73 combined.

Zhou et al. conducted a SEER retrospective review of 6482 patients age ≥ 65 with early stage, triple negative breast cancer (TNBC) [15]. They created a nomogram to determine which patients can avoid chemotherapy by analyzing breast cancer specific survival and overall survival. Predictors used include age, marital status, receipt of radiation, BCS, and tumor characteristics. While this study still needs validation, the authors found that the patients in the high-risk group were noted to have an overall survival and breast cancer-specific survival benefit (both *p* <0.001) from chemotherapy, but those in the low-risk group did not, suggesting it may be a useful tool in determining which older patients with TNBC can safely omit chemotherapy.

### 3.3. Prognostic Models for Patients Having Surgery

Coradini et al. conducted a single institution retrospective study for women age ≥ 70 with non-metastatic disease to explore the predictive ability of pathologic specimen tumor expression of the biological markers ER, PR, pS2 protein, and cathepsin D (CathD) for post-surgical disease-free survival [16]. They used data from 83 patients at a single institution. They found the most predictive model was multivariate using PR, pS2, and CathD, and an internal validation of this model used Harrell’s C statistic to measure goodness of fit of the predicted survival for actual survival. The C statistic was 0.754, which indicates a satisfactory model.

Another model predicting post-surgical outcomes for patients age ≥ 70 was developed by Lemij et al. using data from 547 patients in the prospective multicenter Dutch CLIMB (Climb Every Mountain) cohort [17]. This model predicts the likelihood of developing postoperative complications, such as wound infection or seroma formation, in patients with non-metastatic breast cancer to allow for more tailored preoperative conversations. The model includes five predictive factors. Both internal and external validation were performed. External validation was conducted with data from 2727 patients in the British Bridging the Age Gap Study. The model demonstrated good discrimination in both, with an AUC of 0.76 for internal validation and 0.70 for external validation.

### 3.4. Prognostic Models for Patients on Endocrine Therapy

Soubeyran et al. examined expression of ER, PR, pS2, c-*erb*B-2, and glutathione S-transferase π on core needle biopsies as potential markers to predict tumor regression in response to tamoxifen as neoadjuvant treatment [18]. Data from 208 postmenopausal patients from a single center were utilized. The authors developed a multivariable model using the markers associated with tumor regression, ER, and pS2. This model stratifies patients into low-, intermediate-, and high-risk groups, with response rates of 60%, 45%, and 8%, respectively. This model has not been validated.

Moreau-Bachelard et al. developed the Clinical Treatment Score post-5 years (CTS5), a prognostic tool that predicts the risk of distant recurrence after 5 years of treatment with tamoxifen for early breast cancer [19]. The CTS5 is intended for use to guide decision making surrounding extended use of adjuvant aromatase inhibitors (AI). They developed this model using data from 1105 postmenopausal patients in the Base d’Evaluation et de Recherche des Néoplasmes Infiltrants et in Situ (BERENIS) registry. The model uses age, tumor grade, tumor size, and number of lymph nodes involved with macrometastases to generate a score. The authors state that the model was validated internally but did not provide any validation statistics. They recommend using this tool in combination with bone mineral density, comorbidities, and patient motivation to make decisions about extending tamoxifen therapy.

### 3.5. Prognostic Models to Determine Frailty of Patients

We identified only one study specifically aimed at predicting patient frailty. Brouwers et al. designed a model to examine frailty markers, which was extracted from a single institution’s retrospective data [20]. The study included 162 patients aged ≥70 years old with non-metastatic breast cancer in which the predictive value of aging biomarkers for clinical frailty, defined using their novel 10-point Leuven Oncogeriatric Frailty Score was examined. In these older patients, the only biomarker found to be a useful predictive tool was plasma interleukin-6 (IL-6) levels. There was no validation.

### 3.6. Prognostic Models to Determine the Risk of Mortality in Patients

Our review identified six models to predict survival of older patients with breast cancer. Four of the models were developed to predict both breast cancer-specific death (BCSD) and other cause-specific death (OCSD), one predicted overall mortality and OCSD, and one was limited to BCSD. Only one study is lacking validation.

Fleming et al. developed the Comprehensive Prognosis Index (CPI), which includes two models to predict one-year BCSD and one-year OCSD [21]. Data from 848 patients ≥ 67 years old in the Kentucky Cancer Registry was used to develop the CPI, which used a multiplicative comorbidity index (including 13 comorbidities), age, and cancer stage as predictive factors. Internal validation using actual one-year survival for included patients was performed, which found the observed survival mostly fell within the 95% confidence intervals of CPI-predicted BCSD and OCSD.

The next tool to predict BCSD was a nomogram developed by Lu et al., which predicts 5-, 8-, and 10-year survival for non-metastatic breast cancer [22]. This was developed using data from 20,798 patients, age ≥ 65 years old from the SEER database. Ten independent prognostic factors for BCSD were identified for the nomogram. Internal validation and external validation using data from an additional 4443 patients from SEER were performed. The nomogram showed a high predictive ability in both, with C-indexes of 0.818 (0.804–0.831) and 0.808 (0.783–0.833), respectively.

The Age Gap prognostic model is a nomogram predicting two- and five-year BCSD and OCSD developed by Ward et al. using data from 23,842 women aged ≥70 years with early ER-positive breast cancer from the West Midlands Cancer Intelligence Unit and Northern and Yorkshire Cancer Registry and Information Service registries in the UK [23]. The nomogram incorporates nine predictive factors for BCSD and three for OCSD. This nomogram was internally validated and externally validated using data from 14,562 patients from the Eastern Cancer Registration and Information Centre. Internal and external validation showed that predicted BCSD and OCSD differed from observed rates by <1% and <1.5%, respectively. Interestingly, this model was designed specifically for utilization in the decision to undergo surgery or elect for endocrine therapy only. Treatment is included as one of the 9 covariates in BCSD nomogram, which allows for the generation of a hazard ratio for the 2 treatment options for individual patients.

Peng et al. developed another nomogram to predict both BCSD and OCSD using data from 420 women aged ≥70 years at a single institution [24]. These nomograms predict 1-, 3-, and 5-year survival utilizing 10 prognostic factors. Only internal validation was performed, but the reported C-indexes showed a high predictive ability for both BCSD (0.714) and OCSD (0.717).

Our review includes a unique predictive model by Vargas et al. who measured the predictive ability of systemic inflammatory markers for BCSD [25]. They utilized data from 148 patients ≥ 70 years old treated for node-positive breast cancer at a single institution. Five predictive factors for BCSD were identified. However, no validation was performed.

Finally, the PORTRET (from the prediction of outcome, risk of toxicity and quality of life in older patients treated for breast cancer [the PORTRET] study) tool was developed by Van der Plas-Krigsman et al. to predict not only five-year overall mortality and OCSD, but also recurrence [26]. The model was developed with data from 2744 women age ≥ 70 years in the FOCUS (Female breast cancer in the elderly; Optimizing Clinical guidelines USing clinicopathological and molecular data) retrospective cohort in the Netherlands. Thirteen predictors were used for mortality and OCSD prediction, and eight were utilized for recurrence. Internal and external validation was performed, using data from 13,631 patients in the Netherlands Cancer Registry, by examining discrimination. Internal validation showed the AUCs were 0.76 for overall mortality, 0.78 for OCSD, and 0.73 for recurrence. External validation showed the AUCs were 0.76 for overall mortality, 0.75 for OCSD, and 0.76 for recurrence. Overall, this supports the idea that PORTRET is a highly discriminative tool.

### 3.7. Risk of Bias and Applicability of the Included Studies

The PROBAST was used to examine the risk of bias (ROB) of all studies. Eleven (of the models developed) were assessed as having a low ROB. All models were at low ROB for predictors and outcomes. In model development studies, 14 (82%) models were at low ROB for participants and analysis. The full risk of bias assessment table can be found in Table 3.

The primary issues with the participants domain included using a limited or non-representative population (66%) or too few participants (33%). For the analysis domain, all models with high ROB were found to have incomplete evaluations of their performances.

This PROBAST tool was also used to determine applicability of the included studies, all of which were deemed highly applicable in the current body of evidence for the field.

## 4. Discussion

This study reviewed 17 articles focused on the development and/or validation of prognostic models for older patients with breast cancer. To our knowledge, this is the most comprehensive review of prognostic models specifically made for older patients with breast cancer.

We found that most studies were at low risk of bias. This is a departure from other systematic reviews of prognostic tools for breast cancer [27,28]. Though, it should be noted, these previous studies reported on prognostic tools for patients of all ages, not only older patients. By limiting our search to studies of tools that were specifically designed for older patients, we may have been able to uncover a higher proportion of quality studies due to the inclusion of more specialized investigators, more homogenous patients, and/or more mortality data available.

In our review, the most commonly used predictive factors were age, followed by hormone receptor status and comorbidities then tumor grade. Interestingly, age was not the most common predictive factor in multiple systematic reviews of prognostic tools in patients of all ages [29,30]. This may suggest that age plays an increasingly important role in breast cancer prognosis in older patients, even though the range of ages in the group is fairly limited. We found that the C-index and AUC were the most commonly used strategies in validation, which has been reported in systematic reviews across clinical fields [28]. There is considerable support for these strategies in the literature [31], and this review demonstrates the ease of interpretation of these statistics.

Two of the most promising tools presented in this review are CARG-BC [14] and PORTRET [26]. Both studies have been externally validated and have a low risk of bias. CARG-BC provides clinically relevant information by allowing patients and providers to more fully understand the potential risk and benefit of chemotherapy. This tool only utilizes eight predictors, making its calculation less burdensome. At the same time, it manages to incorporate specific details of the individual chemotherapy plan. While requiring the use of a few more predictor variables (13 total), PORTRET is a highly advantageous tool, as it provides a prediction for not only overall survival, but also risk of recurrence. While including more advanced tumor details than most tools, it also includes predictors that are uniquely prevalent in older patients, like polypharmacy, sensory handicap, and dementia. We advocate for widespread clinical implementation of these tools.

CARG-BC is a derivative of the CARG [32] toxicity tool initially developed to predict risk of high-level chemotherapy toxicity in older patients (age ≥ 65 years) with any type of cancer. Given its more general nature, this model utilizes 11 predictors, rather than 8, highlighting the advantage of tools specifically designed for older patients with breast cancer.

CARG is one of multiple general prognostic tools that were not developed specifically for older patients with breast cancer but are often applied to this population. Others include PREDICT and ePrognosis. PREDICT was initially developed for women of any age with breast cancer to predict overall survival at 5 and 10 years as well as expected benefits of chemotherapy, endocrine treatment, and trastuzumab, but has been validated for prediction of 5-year survival in older patients [33]. ePrognosis is an even more general tool used to predict two-year mortality in patients 70 and older without severe illness, including cancer, and it has been validated specifically for breast cancer [34]. Since these studies have been validated for use in older patients with breast cancer, they are also of use to physicians and older breast cancer patients for clinical decisions, and they can be used in combination with the tools included in this review.

Another interesting study was by Lemij et al., which reports on a model to predict the development of postoperative complications [17], a problem that is especially pertinent to the surgical management of older patients with breast cancer, although mortality from breast cancer related surgery is generally quite low [35]. However, this study had a high ROB due to participant selection. If a more representative group of patients could be studied, clinical care would likely benefit from this tool. This is also true for other interventions, such as radiation and endocrine therapy, that were not found to have high-quality, externally validated tools.

## 5. Limitations

Our systematic review provides a complete overview of available prognostic tools for elderly women with breast cancer according to the CHARMS and PROBAST tools. However, it does have some limitations. Only studies published in English were included. This led to the exclusion of two studies. Our study may also be impacted by publication bias [27]. While inter-rater reliability was high for abstract screening, with a proportionate agreement of 0.985, it was somewhat lower for full text review, with a proportionate agreement of 0.65833. However, any disagreement was usually due to a misunderstanding, and all were definitively resolved. Finally, most of the studies in this review were retrospective in nature, which often introduces bias. In addition, outcomes may have been worse than the investigators in these studies predicted since patients with missing data in large registries, such as SEER, have been shown to have worse outcomes [36].

## 6. Conclusions

We reviewed the development and validation of 17 models predicting response to treatment (including adjuvant radiation, chemotherapy, endocrine therapy, and surgery), frailty, and mortality specific to older patients with breast cancer. There is a wide variety of practices in the development and validation of these models, including the size of the studies, inclusion criteria, patient demographics, predictive factors included, and analytic strategies used. While external validation was rare (only performed for five studies), we found that they were concordant with interval validation results. External validation was also most common for models predicting mortality. Studies that were not validated or only internally validated need external validation. However, most of the models presented in this review represent promising tools for clinical application in the care of older patients with breast cancer.

## Figures and Tables

**Table 1:** Baseline characteristics of tools included in a systematic review of prognostic tools for older patients with breast cancer [10,11,12,13,14,15,16,17,18,19,20,21,22,23,24,25,26].

Tool	Authors	Year of Publication	Minimum Patient Age	Number of Patients in Development Cohort	Source of Data for Development Cohort	Number of Patients in Validation Cohort *	Source of Data for Validation Cohort *
Nomogram for benefit from RT	Albert et al. [10]	2012	66	16,092	SEER		
Nomogram for benefit from RT	Chen et al. [11]	2015	70	9079	SEER		
Radiation for Older Women	Wang et al. [12]	2020	65	0	56,700 simulations		
Tool for spinal metastasis benefit from RT	Rades et al. [13]	2015	65	218	University of Lübeck, Germany		
Cancer and Aging Research Group-Breast Cancer score	Magnuson et al. [14]	2021	65	283	HOPE	190	HOPE
Nomogram for omitting chemotherapy in TNBC	Zhou et al. [15]	2022	65	6482	SEER		
Tumor markers for post-surgical DFS	Coradini et al. [16]	1998	70	83	National Tumor Institute, Italy		
Model for postoperative complications	Lemij et al. [17]	2022	70	547	CLIMB	2727	Bridging the Age Gap Study
Tumor markers for response to neoadjuvant Tamoxifen	Soubeyran et al. [18]	1996	postmenopausal	208	Bergonie Institue, France		
Clinical Treatment Score post-5 years	Moreau-Bachelard et al. [19]	2020	postmenopausal	1105	BERENIS		
Biomarkers for frailty	Brouwers et al. [20]	2015	70	162	University Hospitals Leuven		
Comprehensive Prognosis Index	Fleming et al. [21]	1999	67	848	Kentucky Cancer Registry		
Nomogram for survival	Lu et al. [22]	2020	65	20,798	SEER	4443	SEER
Age Gap	Ward et al. [23]	2020	70	23,842	West Midlands Cancer Intelligence Unit and Northern and Yorkshire Cancer Registry and Information Service registries, UK	14,562	Eastern Cancer Registration and Information Centre
Nomogram for survival	Peng et al. [24]	2021	70	420	Peking University People’s Hospital, China		
Inflammatory markers for survival	Vargas et al. [25]	2022	70	148	Diego Portales University, Chile		
PORTRET	Van der Plas-Krigsman et al. [26]	2021	70	2744	FOCUS	13,631	Netherlands Cancer Registry

Abbreviations: RT = radiation therapy; TNBC = triple negative breast cancer; SEER = Surveillance, Epidemiology, and End Results Program; HOPE = Hurria Older PatiEnts (HOPE) with Breast Cancer Cohort Study; DFS = disease-free survival; CLIMB = Dutch CLIMB (Climb Every Mountain) cohort; BERENIS = Base d’Evaluation et de REcherche des Néoplasmes Infiltrants et in Situ (France); PORTRET = prediction of outcome, risk of toxicity and quality of life in older patients treated for breast cancer; FOCUS = Female breast cancer in the elderly; Optimizing Clinical guidelines USing clinicopathological and molecular data. * “Validation cohort” refers to an external validation cohort. This column was left blank if a tool has not been externally validated, though authors may have performed internal validation.

**Table 2:** Prediction details of tools included in a systematic review of prognostic tools for older patients with breast cancer [10,11,12,13,14,15,16,17,18,19,20,21,22,23,24,25,26].

Tool	Authors	Eligibility Criteria	Outcome Predicted	Number of Predictive Factors	Predictive Factors
Nomogram for benefit from RT	Albert et al. [10]	any unilateral breast cancer treated with BCS	mastectomy-free survival benefit from adjuvant RT	6	age, race, tumor size, ER status, RT, nodal pathology
Nomogram for benefit from RT	Chen et al. [11]	stage I & ER- or stage II/III breast cancer treated with BCS	BCSS benefit from adjuvant RT	6	ER status, PR status, tumor grade, T stage, N stage, race
Radiation for Older Women	Wang et al. [12]	early stage, ER+, LN-negative breast cancer treated with BCS	OS and local recurrence benefit from adjuvant RT	16	age, BMI, cigarette use, COPD, other cancer, CJF, DM, difficulty walking several blocks, difficult managing finances, difficulty bathing, difficulty pushing/pulling large objects, tumor grade, tumor size, ER status, margin, LN, additional health conditions, additional breast cancer factors
Tool for spinal metastasis benefit from RT	Rades et al. [13]	any breast cancer metastasized to the spinal cord	OS benefit from spinal RT	9	fractionation regimen, age, time from breast cancer diagnosis to RT of MSCC, visceral metastases, other bone metastases, time developing motor deficits, pre-radiotherapy ambulatory status, number of involved vertebrae, and Eastern Cooperative Oncology Group (ECOG) performance score
Cancer and Aging Research Group-Breast Cancer score	Magnuson et al. [14]	stage I–III breast cancer	likelihood of grade 3–5 chemotherapy toxicity	8	stage, use of anthracyclines, treatment duration, hemoglobin, liver function, number of falls in last 6 months, ability to walk 1 mile, present of support person for crisis
Nomogram for omitting chemotherapy in TNBC	Zhou et al. [15]	T1-2, N0-1, M0 TNBC	OS and BCSS benefit from chemotherapy	7	age, married status, grade, T-stage, N-stage, RT, BCS
Tumor markers for post-surgical DFS	Coradini et al. [16]	any M0 breast cancer treated with surgery	post-surgical DFS	3	PR, pS2, cathepsin D
Model for postoperative complications	Lemij et al. [17]	any M0 breast cancer	likelihood of post-surgical complication	5	age, polypharmacy, BMI, type of breast surgery, type of axillary surgery
Tumor markers for response to neoadjuvant Tamoxifen	Soubeyran et al. [18]	any M0 breast cancer	tumor regression in repones to tamoxifen	2	ER, pS2
Clinical Treatment Score post-5 years	Moreau-Bachelard et al. [19]	early, ER+ breast cancer treated with ≥4.5 years of tamoxifen	risk of distance recurrence after 5 years of tamoxifen	4	tumor grade, age, tumor size, number of LN involved
Biomarkers for frailty	Brouwers et al. [20]	any M0 breast cancer	Leuven Oncogeriatric Frailty Score	1	plasma IL-6
Comprehensive Prognosis Index	Fleming et al. [21]	any breast cancer	1-year BCSD and OCSD	15	age, cancer stage, acute MI/unstable angina, CHF, cerebrovascular disease/stroke, dementia, hemiplegia/paraplegia, other neurologic disorder, mild-moderate pulmonary disease, mild-moderate renal disease, severe ESRD, mild-moderate gallbladder/pancreas disease, severe gallbladder/pancreas disease, any other cancer, coagulopathy
Nomogram for survival	Lu et al. [22]	any M0 breast cancer	5-, 8-, and 10-year BCSD	10	age, marital statis, race, IDC vs. ILC, grade, T stage, N stage, ER, PR, surgery type
Age Gap	Ward et al. [23]	ER+ early breast cancer	2- and 5-year BCSD and OCSD	9	primary surgical vs. endocrine treatment, age, Charlson co-morbidity index, frailty score, grade, nodal status, size, detected on screening vs. symptomatic, deprivation rating of postal code
Nomogram for survival	Peng et al. [24]	any breast cancer	1-, 3-, and 5-year BCSD and OCSD	10	age, treatment with chemotherapy, number of comorbidities, HR, HER2, Ki67, T stage, N stage, receipt of surgery, receipt of RT
Inflammatory markers for survival	Vargas et al. [25]	any node-positive breast cancer	risk of BCSD	5	levels of monocytes, levels of neutrophils, neutrophil-to-lymphocytes ratio, level of eosinophils, eosinophil multiple by neutrophils-to-lymphocytes ratio
PORTRET	Van der Plas-Krigsman et al. [26]	operable breast cancer treated with locoregional therapy	5-year overall mortality, 5-year OCSD, and risk of recurrence	13	age, size, nodal status, grade, HR, HER2, Ki67, number of comorbidities, polypharmacy, difficulty walking, sensory handicap, dementia

Abbreviations: RT = radiation therapy; TNBC = triple negative breast cancer; ER = estrogen receptor; PR = progesterone receptor; LN = lymph node; BCS = breast-conserving surgery; BCSS = breast cancer-specific survival; OS = overall survival; BCSD = breast cancer-specific death; OCSD = other-cause-specific death; BMI = body mass index; COPD = chronic obstructive pulmonary disease; CHF = congestive heart failure; DM = diabetes mellitus; MSCC = metastatic spinal cord cancer; DFS = disease-free survival; IDC = invasive ductal carcinoma; ILC = invasive lobular carcinoma; PORTRET = prediction of outcome, risk of toxicity and quality of life in older patients treated for breast cancer.

**Table 3:** Risk of bias of tools included in a systematic review of prognostic tools for older patients with breast cancer [10,11,12,13,14,15,16,17,18,19,20,21,22,23,24,25,26].

Study	Participants Selection	Predictor Factor Measurement	Outcome Measurement	Statistical Analysis	Overall Risk of Bias
Albert et al. [10]	low	low	low	low	low
Chen et al. [11]	low	low	low	low	low
Wang et al. [12]	unclear	low	low	low	high
Rades et al. [13]	low	low	low	high	high
Magnuson et al. [14]	low	low	low	low	low
Zhou et al. [15]	low	low	low	low	low
Coradini et al. [16]	low	low	low	low	low
Lemij et al. [17]	high	low	low	low	high
Soubeyran et al. [18]	low	low	low	high	high
Moreau-Bachelard et al. [19]	low	low	low	low	low
Brouwers et al. [20]	low	low	low	low	low
Fleming et al. [21]	low	low	low	low	low
Lu et al. [22]	high	low	low	low	high
Ward et al. [23]	low	low	low	low	low
Peng et al. [24]	low	low	low	low	low
Vargas et al. [25]	low	low	low	high	high
Van der Plas-Krigsman et al. [26]	low	low	low	low	low

## Data Availability

No new data were created or analyzed in this study. Data sharing is not applicable to this article.

## Appendix A. Search Strategy Report for a Systematic Review of Prognostic Tools for Older Patients with Breast Cancer

Topic: What risk-estimation tools are best to use in elderly patients with breast cancer to prevent over- or under-treatment?

Searcher: S.J.K.

Date: 11 August 2022

**Table A1:** Database (including vendor/platform): MEDLINE (via PubMed).

Set #	Search Strategy	Results
#1 Elderly	“Aged”[Mesh] OR “Health Services for the Aged”[Mesh] OR Aged[tiab] OR aging[tiab] OR older[tiab] OR oldest[tiab] OR senior[tiab] OR seniors[tiab] OR Geriatrics[tiab] OR geriatric[tiab] OR Elderly[tiab] OR elder[tiab] OR elders[tiab] OR centenarian[tiab] OR centenarians[tiab] OR nonagenarian[tiab] OR nonagenarians[tiab] OR octogenarian[tiab] OR octogenarians[tiab]	4,320,864
#2 Breast Cancer	“Breast Neoplasms”[Mesh] OR ((breast[tiab] OR breasts[tiab] OR mammary[tiab] OR lobular[tiab]) AND (cancer[tiab] OR cancers[tiab] OR neoplasm[tiab] OR neoplasms[tiab] OR carcinoma[tiab] OR carcinomas[tiab] OR tumor[tiab] OR tumors[tiab] OR tumour[tiab] OR tumours[tiab] OR malignant[tiab] OR malignancy[tiab] OR malignancies[tiab] OR neoplasia[tiab] OR cancerous[tiab]))	492,408
#3 Tools	Tool[tiab] OR tools[tiab] OR instrument[tiab] OR instruments[tiab] OR PORTRET[tw] OR RSClin[tw] OR NSQIP[tw] OR “CARG-TT”[tw] OR CRASH[tw] OR “CARG-BC”[tw]	1,119,339
#4 Patients	“patients”[mesh] OR Patient[tiab] OR patients[tiab] OR woman[tiab] OR women[tiab]	8,628,783
#5	#1 AND #2 AND #3 AND #4	3655
Validation string	23389352 OR 7091475 OR 16904902 OR 22433762 OR 11786579 OR 20308657 OR 25071125 OR 27048496 OR 36098027 OR 33444080 OR 22734034	5/11
	Exemplar articles retrieved: 1: Magnuson A, Sedrak MS, Gross CP, Tew WP, Klepin HD, Wildes TM, Muss HB, Dotan E, Freedman RA, O’Connor T, Dale W, Cohen HJ, Katheria V, Arsenyan A, Levi A, Kim H, Mohile S, Hurria A, Sun CL. Development and Validation of a Risk Tool for Predicting Severe Toxicity in Older Adults Receiving Chemotherapy for Early- Stage Breast Cancer. J Clin Oncol. 2021 Feb 20;39(6):608–618. doi:10.1200/JCO.20.02063. Epub 2021 Jan 14. PMID: 33444080; PMCID: PMC8189621. [14] 2: van der Plas-Krijgsman WG, Giardiello D, Putter H, Steyerberg EW, Bastiaannet E, Stiggelbout AM, Mooijaart SP, Kroep JR, Portielje JEA, Liefers GJ, de Glas NA. Development and validation of the PORTRET tool to predict recurrence, overall survival, and other-cause mortality in older patients with breast cancer in the Netherlands: a population-based study. Lancet Healthy Longev. 2021 Nov;2(11):e704-e711. doi: 10.1016/S2666-7568(21)00229-4. Epub 2021 Nov 3. PMID:36098027. [26] 3: Kanatas A, Velikova G, Roe B, Horgan K, Ghazali N, Shaw RJ, Rogers SN. Patient-reported outcomes in breast oncology: a review of validated outcome instruments. Tumori. 2012 Nov;98(6):678–88. doi: 10.1177/030089161209800602. PMID: 23389352. 4: Albert JM, Liu DD, Shen Y, Pan IW, Shih YC, Hoffman KE, Buchholz TA, Giordano SH, Smith BD. Nomogram to predict the benefit of radiation for older patients with breast cancer treated with conservative surgery. J Clin Oncol. 2012 Aug 10;30(23):2837–43. doi: 10.1200/JCO.2011.41.0076. Epub 2012 Jun 25. PMID: 22734034; PMCID: PMC3410401. [10] 5: Maratia S, Cedillo S, Rejas J. Assessing health-related quality of life in patients with breast cancer: a systematic and standardized comparison of available instruments using the EMPRO tool. Qual Life Res. 2016 Oct;25(10):2467–2480. doi: 10.1007/s11136-016-1284-8. Epub 2016 Apr 5. PMID:27048496.	

**Table A2:** Database (including vendor/platform): Embase.

Set #	Search Strategy	Results
#1 Elderly	‘aged’/exp OR ‘elderly care’/exp OR Aged:ti,ab OR aging:ti,ab OR older:ti,ab OR oldest:ti,ab OR senior:ti,ab OR seniors:ti,ab OR Geriatrics:ti,ab OR geriatric:ti,ab OR Elderly:ti,ab OR elder:ti,ab OR elders:ti,ab OR centenarian:ti,ab OR centenarians:ti,ab OR nonagenarian:ti,ab OR nonagenarians:ti,ab OR octogenarian:ti,ab OR octogenarians:ti,ab	4,909,255
#2 Breast Cancer	‘breast tumor’/exp OR ((breast:ti,ab OR breasts:ti,ab OR mammary:ti,ab OR lobular:ti,ab) AND (cancer:ti,ab OR cancers:ti,ab OR neoplasm:ti,ab OR neoplasms:ti,ab OR carcinoma:ti,ab OR carcinomas:ti,ab OR tumor:ti,ab OR tumors:ti,ab OR tumour:ti,ab OR tumours:ti,ab OR malignant:ti,ab OR malignancy:ti,ab OR malignancies:ti,ab OR neoplasia:ti,ab OR cancerous:ti,ab))	768,102
#3 Tools	Tool:ti,ab OR tools:ti,ab OR instrument:ti,ab OR instruments:ti,ab OR PORTRET:ti,ab OR RSClin:ti,ab OR NSQIP:ti,ab OR ‘CARG TT’:ti,ab OR CRASH:ti,ab OR ‘CARG BC’:ti,ab	1,482,656
#4 Patients	‘patient’/exp OR Patient:ti,ab OR patients:ti,ab OR woman:ti,ab OR women:ti,ab	13,110,015
#5	#1 AND #2 AND #3 AND #4	5276
#6	#1 AND #2 AND #3 AND #4 AND [humans]/lim	5208
#7	#1 AND #2 AND #3 AND #4 AND [humans]/lim AND ([article]/lim OR [article in press]/lim OR [conference paper]/lim OR [review]/lim)	4294

**Table A3:** Database (including vendor/platform): Web of Science Core Collection (1900–present).

Set #	Search Strategy	Results
#1 Elderly	TS = (Aged OR aging OR older OR oldest OR senior OR seniors OR Geriatrics OR geriatric OR Elderly OR elder OR elders OR centenarian OR centenarians OR nonagenarian OR nonagenarians OR octogenarian OR octogenarians)	5,230,284
#2 Breast Cancer	TS = ((breast OR breasts OR mammary OR lobular) AND (cancer OR cancers OR neoplasm OR neoplasms OR carcinoma OR carcinomas OR tumor OR tumors OR tumour OR tumours OR malignant OR malignancy OR malignancies OR neoplasia OR cancerous))	713,188
#3 Tools	TS = (Tool OR tools OR instrument OR instruments OR PORTRET OR RSClin OR NSQIP OR CARG-TT OR CRASH OR CARG-BC)	2,594,136
#4 Patients	TS = (Patient OR patients OR woman OR women)	8,611,148
#5	#1 AND #2 AND #3 AND #4	3612
